# Interaction between Amyloid Beta Peptide and an Aggregation Blocker Peptide Mimicking Islet Amyloid Polypeptide

**DOI:** 10.1371/journal.pone.0020289

**Published:** 2011-05-25

**Authors:** Nasrollah Rezaei-Ghaleh, Erika Andreetto, Li-Mei Yan, Aphrodite Kapurniotu, Markus Zweckstetter

**Affiliations:** 1 Department for NMR-based Structural Biology, Max-Planck-Institute for Biophysical Chemistry, Göttingen, Germany; 2 Division of Peptide Biochemistry, Technische Universität München, Freising, Germany; 3 DFG Research Center for the Molecular Physiology of the Brain (CMPB), Göttingen, Germany; University of Medicine and Dentistry of New Jersey, United States of America

## Abstract

Assembly of amyloid-beta peptide (Aβ) into cytotoxic oligomeric and fibrillar aggregates is believed to be a major pathologic event in Alzheimer's disease (AD) and interfering with Aβ aggregation is an important strategy in the development of novel therapeutic approaches. Prior studies have shown that the double N-methylated analogue of islet amyloid polypeptide (IAPP) IAPP-GI, which is a conformationally constrained IAPP analogue mimicking a non-amyloidogenic IAPP conformation, is capable of blocking cytotoxic self-assembly of Aβ. Here we investigate the interaction of IAPP-GI with Aβ40 and Aβ42 using NMR spectroscopy. The most pronounced NMR chemical shift changes were observed for residues 13–20, while residues 7–9, 15–16 as well as the C-terminal half of Aβ - that is both regions of the Aβ sequence that are converted into β-strands in amyloid fibrils - were less accessible to solvent in the presence of IAPP-GI. At the same time, interaction of IAPP-GI with Aβ resulted in a concentration-dependent co-aggregation of Aβ and IAPP-GI that was enhanced for the more aggregation prone Aβ42 peptide. On the basis of the reduced toxicity of the Aβ peptide in the presence of IAPP-GI, our data are consistent with the suggestion that IAPP-GI redirects Aβ into nontoxic “off-pathway” aggregates.

## Introduction

Protein misfolding and aggregation into amyloid fibrillar aggregates are associated with a number of severe neurodegenerative diseases including Alzheimer's disease (AD) [1]. The main pathological hallmarks of AD are the extracellular “senile plaques”, constituted of amyloid β (Aβ) peptide, and the intracellular “neurofibrillary tangles” of tau protein [2]. Several genetic and pathologic evidences support the “amyloid cascade hypothesis” of AD, according to which Aβ peptide self-association plays a vital initiating role in AD pathogenesis, both in familial and sporadic forms of disease [3], [4]. Aβ peptide constitutes a small part of a large transmembrane protein, the amyloid precursor protein (APP), and is released to the extracellular environment after two consecutive proteolytic cleavages of APP by β- and γ-secretase [5]. The 39–42 residue Aβ peptide is mostly unstructured in aqueous solution [6]–[8], but has a tendency to undergo a conformational transition to β-sheet aggregates [9]. This self-association of Aβ is known to exert its neurotoxic effects [10]. While the final amyloid fibrillar state of Aβ was originally believed to cause the main toxic effects of this peptide, increasing evidence suggests that the earliest and most extensive cytotoxic properties of Aβ are mediated by smaller less ordered assemblies of Aβ [4], [11], [12]. Accordingly, Aβ aggregation-based strategies to search for AD-modifying drugs should be targeted to prevent buildup of any toxic oligomeric species along the assembly reaction.

**Figure 1:**
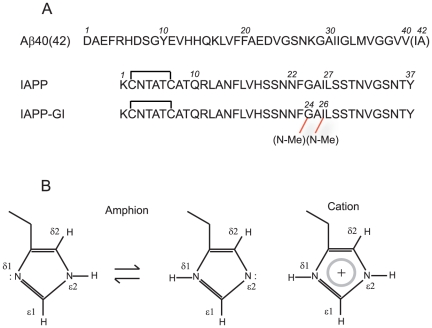
Primary structures of Aβ, IAPP and IAPP-GI (A) and tautomeric and protonation states of histidine side chain (B). ^1^H-^15^N HSQC spectra of histidine side chains were acquired with coherence transfer from ε1H and δ2H to ε2N and δ1N through ^2^J coupling constants.

One approach to inhibit an aggregation reaction is to re-design the self-recognition interface of peptide or protein molecules, in a manner that the modified molecule is still capable of interacting with the native form, but inhibits its further assembly into larger aggregates [13]. This approach was successfully exploited for example in the case of Islet Amyloid Polypeptide (IAPP) [13], [14]. IAPP is a hydrophobic strongly amyloidogenic peptide, which is produced in the pancreatic islet cells and plays, in the soluble monomeric form, a major role in glucose homeostasis [15]. In certain circumstances, this peptide undergoes a transition from a predominantly random coil monomeric state to β-sheet aggregates, and these aggregates have been shown to be strongly toxic for several cell types and associated with the progressive destruction of pancreatic beta cells in adult-onset diabetes [16]. To keep the physiologic function of IAPP but block its amyloid forming propensity, an IAPP analogue was designed through the structure-based introduction of a minimum number of two N-methyl groups, at Gly24 and Ile26, located in the amyloid core and with side chains presumably pointing into a similar direction (see Figure 1A) [13], [17]. It was shown that the so-called IAPP-GI peptide efficiently inhibits IAPP amyloid formation and cytotoxicity [13], [17]. More interestingly, IAPP-GI was demonstrated to block cytotoxic assembly of Aβ and insulin as well [18], [19]. The cross-association reaction between IAPP-GI (or nonfibrillar IAPP conformers) and Aβ may have some implications beyond its therapeutic potentials, and along with clinical and epidemiological evidences, provide a potential molecular link between AD and adult-onset diabetes [20]. On a more general level and based on recent evidence for other cross-interactions between amyloidogenic polypeptides and proteins including the Aβ-tau and the Aβ-prion protein interaction, it appears that cross-amyloid interactions may play a critical role in neurodegenerative diseases [21], [22].

In the current study, we make use of solution Nuclear Magnetic Resonance (NMR) measurements to investigate the intermolecular interaction of Aβ with IAPP-GI. Considering the high sequence similarity of Aβ and IAPP-GI and the blockade of Aβ aggregation by IAPP-GI, the results of this study may assist in understanding the mechanism of the very early steps of Aβ self-association into cytotoxic aggregates and fibrils and its inhibition.

## Results and Discussion

Both Aβ and IAPP peptides show a remarkable hydrophobicity in the sequence and a high tendency to self-associate, especially at concentrations which are normally used in NMR experiments. Accordingly, Aβ-IAPP hetero-association may encounter significant kinetic and/or thermodynamic barriers if peptide self-association is a competing alternative scenario for them. Therefore, we needed to find an experimental condition under which these two peptides predominantly populate a monomeric state. Several studies demonstrate that the full-length Aβ40 is mostly monomeric at neutral pH [8], [23], and the peptide does not significantly change the assembly state if the sample is stored at concentrations below 100 µM and temperatures as low as 5°C, provided that the sample is devoid of preformed aggregates [8]. IAPP is also known to be much less prone to aggregation at lower temperatures [24], and it has been suggested on the basis of analytical ultracentrifugation and diffusion NMR spectroscopy that human IAPP is in a kinetically stable state consisting of monomers and high molecular weight oligomers at neutral pH and 4°C, even at a concentration as high as 255 µM (1 mg/mL) [24], [25]. However, at temperatures above 20°C, the strong self-association propensity of human IAPP rapidly drives monomeric IAPP molecules toward oligo- and multimerization [24]–[27]. At this temperature, IAPP-GI has also been shown to self-associate -similarly to IAPP- with low nanomolar affinity into soluble di- and oligomers [13]. In contrast to IAPP, however, IAPP-GI oligomers were found to be unable to further aggregate into cytotoxic aggregates and amyloid fibrils, due to the conformational constraint imposed by the two N-methylations [13], [17]. Taking all these points into consideration, we selected as our titration experimental condition a low temperature of 5°C and a neutral pH of 7.3 buffered with 20 mM sodium phosphate without addition of any salt. Aβ40, Aβ42 - that shows a higher aggregation propensity than Aβ40 and is believed to be the primary toxic amyloid β peptide [2] - and IAPP-GI in their lyophilized states were dissolved in 10 mM NaOH and pure HFIP, respectively, as recommended in references [8], [15]. Aβ concentrations were kept constant at 30 µM along the titration experiment, while the IAPP-GI concentration was varied in the range of 0–480 µM [25].

**Figure 2:**
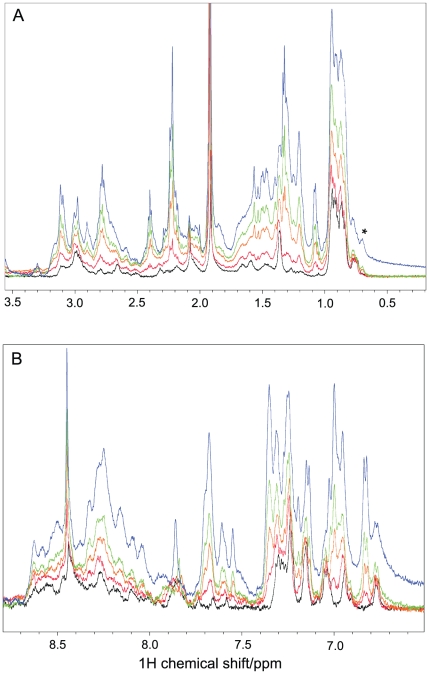
1D ^1^H NMR spectra of Aβ40 with or without IAPP-GI. The aliphatic and amide regions of the spectra are shown in (A) and (B), respectively. The spectra were measured in the presence of 0 (black), 2 (red), 4 (orange), 8 (green) and 16 (blue) molar excess of IAPP-GI. Chemical shifts were calibrated on the basis of an external DSS reference and temperature was 5°C. Note the peak at 0.7 ppm, marked as *, which is probably related to an oligomeric state of IAPP-GI or Aβ40. Peaks at 1.15, 6.82, 7.00 and 7.35 ppm are exclusively observed after addition of IAPP-GI.

The 1D ^1^H-NMR spectra of Aβ40 and Aβ42 in the absence of IAPP-GI are characteristic of unfolded peptides, i.e. methyl resonances are found in two narrow regions of 0.8–1 and 1.3–1.4 ppm, characteristic for random coil shifts of Val, Leu, Ile and Ala (Figures 2 and S1). In agreement with the absence of a threonine residue in the Aβ40 as well as Aβ42 primary sequence, no signal was observed at around 1.1 ppm. After addition of IAPP-GI, the overall signal intensity was increased, and some new signals appeared, e.g. there were two new signals at around 1.1 and 1.2 ppm, i.e. close to the random coil chemical shift of the methyl group of Thr residues, and therefore probably originate from the four Thr residues of IAPP-GI. In addition, NMR signals were observed at 6.82, 7.00 and 7.35 ppm exclusively in the presence of IAPP-GI (Figures 2 and S1). The intensities of the IAPP-GI specific NMR signals represent sensitive probes of the concentration for the NMR-visible IAPP-GI molecules during the titration. In case of Aβ40, the IAPP-GI specific signals increased in intensity by factors of 2.1, 3.6 and 6.8 in the spectra with 4, 8 and 16 molar excess of IAPP-GI when compared to the spectrum with two-fold excess of IAPP-GI. The slower rise in the peak intensity than expected on the basis of the added amount of IAPP-GI suggests that IAPP-GI partially aggregates to large NMR-invisible states during the titration. In case of Aβ42, the relative intensity of the IAPP-GI specific NMR signals increased even less with increasing IAPP-GI concentration, suggesting that the Aβ peptide and its specific aggregation propensity plays a role in aggregation of IAPP-GI (Figure S1). The formation of aggregates during the titration is further supported by the emergence of an additional, relatively broad signal at around 0.7 ppm (marked with *), which appeared at a IAPP-GI∶Aβ40 ratio of 4∶1 and became stronger at the ratio of 16∶1. In case of Aβ42, this broad peak is already present at a IAPP-GI∶Aβ42 ratio of 1∶1. The new NMR signal may be related to an oligomeric state of IAPP-GI in co-existence with the monomeric state, as suggested for IAPP at low temperatures [25], or alternatively be caused by an oligomeric state of Aβ, now induced by a high concentration of IAPP-GI [28].

**Figure 3:**
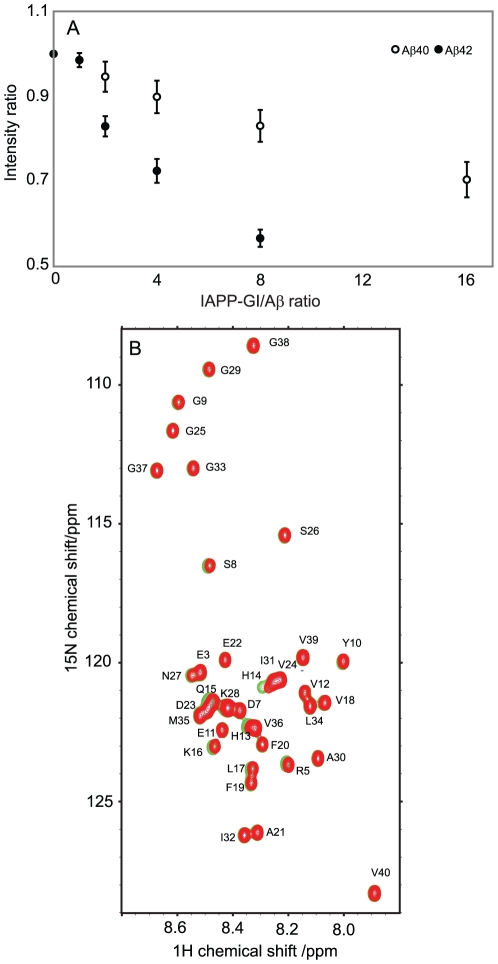
Probing the binding of IAPP-GI to Aβ using NMR spectroscopy. A. Variation of ^1^H-^15^N-HSQC peak intensity of Aβ40 or Aβ42 upon titration with un-labeled IAPP-GI. Intensity ratios were calculated relative to intensities of free Aβ spectra, and error bars represent variation along the sequence. Note that loss of signal intensity is more enhanced for Aβ42 than Aβ40. B. ^1^H-^15^N-HSQC spectra of Aβ40 in the absence (green) or presence of 16 molar excess of non-labeled IAPP-GI (red). A shift in the position of His13, His14, Gln15, Lys16 and Leu17 peaks is evident.

To obtain site-resolved information about the interaction of Aβ with IAPP-GI, a series of two-dimensional ^1^H-^15^N HSQC measurements were performed for 30 µM of ^15^N-labeled Aβ40 and Aβ42 at increasing concentrations of IAPP-GI. Increasing concentrations of IAPP-GI resulted in a uniform reduction of signal intensities of Aβ40 and Aβ42: the average intensity ratio of the backbone signals of Aβ42 was 0.57(±0.02) for the last titration point when compared to the first one (Figures 3A and S2). In addition, the side-chain signals of Arg5, Gln15 and Asn27 obeyed a similar trend of decreasing intensity. The uniform loss of NMR signal intensities in the backbone and side chains upon increasing IAPP-GI concentrations suggests that IAPP-GI induces aggregation of Aβ42 into large oligomeric NMR-invisible states. The bigger loss of Aβ42 intensity when compared to Aβ40 (43 percent *vs* 16 percent at the same IAPP-GI/Aβ ratio) is consistent with the higher aggregation propensity of Aβ42 compared to Aβ40. Together with the aggregation of IAPP-GI in the presence of Aβ described above (Figures 2 and S1), our data reveal a concentration-dependent co-aggregation of Aβ and IAPP-GI that is enhanced for the more aggregation prone Aβ42 peptide.

**Figure 4:**
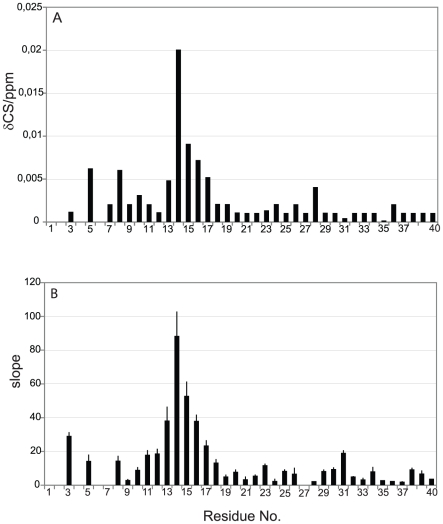
NMR chemical shift perturbation in Aβ40 upon addition of IAPP-GI. A. Average backbone N and H^N^ chemical shift difference of Aβ40 after addition of a 16 fold molar excess of IAPP-GI. B. Slope of the best fitted line to chemical shift perturbation *vs* IAPP-GI concentration data, presented in units of ppm/M.

**Figure 5:**
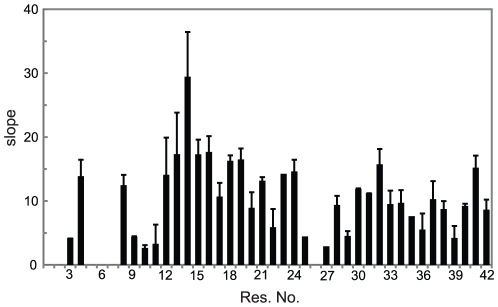
Average backbone N and H^N^ chemical shift perturbation in Aβ42 upon addition of IAPP-GI. The values are reported as slope of the best fitted line to chemical shift difference *vs* IAPP-GI concentration data, in units of ppm/M.

Besides the uniform decrease in NMR signal intensity, several residues showed small changes in their N and H^N^ chemical shifts after addition of IAPP-GI, while no significant chemical shift perturbation was found in the control experiments (Figure 3B). The strongest chemical shift changes were observed in a stretch of the peptide extending from His13 to Phe20, with additional perturbations appearing at residues Arg5, Ser8 and Asp23 (Figure 4A). Moreover, chemical shift changes of most of the peaks followed a linear relationship up to a molar ratio of IAPP-GI∶Aβ40 of 16∶1, with the largest linear slopes observed for residues His13-Lys16 (Figure 4B). Similar chemical shift changes were observed upon addition of IAPP-GI to Aβ42 (Figure 5). The gradual change in chemical shifts, the absence of new peaks and the lack of any site-specific broadening effect during the titration, suggests that the interaction between the two peptides occurs in the fast exchange regime on the NMR time scale and that the affinity of IAPP-GI to bind Aβ is relatively weak at the conditions of the NMR experiment.

At first sight, the low affinity between Aβ and IAPP-GI observed in the NMR experiments appears to be in contrast with the previously reported affinity of their interaction as derived from fluorescence titration assays [18]. In these studies, N-terminal fluorescein labeled IAPP-GI (Fluos-IAPP-GI) (1 nM) was titrated with Aβ40 using low nano- to submicromolar Aβ40 concentrations and an app. K_d_ of 50 nM was determined [18]. Our current results together with those of previous studies suggest, however, that the differences in apparent affinities are due to different peptide assembly states - regarding both Aβ40 and IAPP-GI - present in the NMR titration studies as compared to the fluorescence titration assay [18]. Indeed, at the micromolar peptide concentrations used in the NMR titration study, two oligomeric Aβ40 species of ∼30 and ∼150 nm in hydrodynamic radius were observed using dynamic light scattering (Figure S3 and Methods S1), despite using NaOH to dissolve pre-formed Aβ40 aggregates [8]. Addition of IAPP-GI led then to further aggregation of Aβ40 as shown above. Similar to Aβ40, IAPP-GI populates an aggregate state in equilibrium with the monomeric state, with a shift toward aggregation occurring at higher IAPP-GI concentrations [26]. In fact, the high self-assembly propensity of IAPP-GI into soluble but -in contrast to IAPP- nonamyloidogenic dimers and oligomers, with self-assembly starting already in the low nanomolar concentration range, has been earlier demonstrated using various biophysical methods [13]. These included fluorescence spectroscopy, i.e. titration of Fluos-IAPP-GI (1 nM) with IAPP-GI (app. K_d_, 4 nM) [13], transmission electron microscopy [13], CD concentration dependence studies ([13], see also Figure S4 and Methods S1), size exclusion chromatography [13], and SDS-PAGE and Western blot analysis of cross-linked IAPP-GI samples [18]. Based on the above data, it becomes evident that while the app. K_d_ of the IAPP-GI-Aβ40 interaction measured using fluorescence spectroscopy was determined under conditions where IAPP-GI is mainly in a monomeric state [13] and Aβ40 mainly in a monomer or dimer state (submicromolar concentrations), the interaction followed in our NMR study here included at least in a large part interactions between (NMR-visible) Aβ monomers and IAPP-GI oligomeric assemblies. Therefore, the actual monomeric concentrations of Aβ and IAPP-GI under the NMR conditions were considerably less than the nominal peptide concentrations, and the apparent affinities observed here using NMR are not comparable with those of fluorescence titration assays obtained at nanomolar concentrations of Aβ40 and IAPP-GI. In addition, as both IAPP-GI and Aβ40 (or Aβ42) have been recently shown to use the same regions in their self- and hetero-association processes, a reduced affinity between interactions of their oligomeric states would be expected which is consistent with the NMR observations [38]. At the same time, however, a strong inhibitory effect of IAPP-GI on Aβ40 and Aβ42 fibrillogenesis and cytotoxicity has been shown using assay systems containing low micromolar concentrations of Aβ and IAPP-GI [18], [38], suggesting that our studies are relevant for the understanding of the inhibitory mechanism of IAPP-GI on Aβ. To obtain more direct evidence for the effect of IAPP-GI oligomerization on its binding affinity to Aβ40, we titrated synthetic N-terminal fluorescein labeled Aβ40 (Fluos-Aβ40) (1 nM) with increasing amounts of IAPP-GI (Figure S5 and Methods S1). Binding of IAPP-GI to Fluos-Aβ40 resulted in a strong increase of the fluorescence emission. A sigmoidal binding isotherm was obtained with a plateau at an IAPP-GI concentration between 250–500 nM and curve fitting yielded an app. K_d_ of 39 nM for the Fluos-Aβ40-IAPP-GI interaction of 39 nM (Figure S5A). The obtained K_d_ was nearly identical to the app. K_d_ of 41.2 nM (±3.9) which was obtained by titrating Fluos-IAPP-GI (1 nM) with Aβ40 confirming thus this previously reported high affinity of interaction between the two peptides [18]. However, titrating Fluos-Aβ40 with IAPP-GI using IAPP-GI concentrations higher than 500 nM, i.e. between 1–10 µM, resulted in a dramatic decrease of the fluorescence emission determined at the plateau of the Fluos-Aβ40-IAPP-GI mixture (Figure S5B). These findings were consistent with the suggestions (a) that significant amounts of different IAPP-GI oligomeric assemblies are populated at concentrations higher than 1 µM as compared to the species present between 1 and 500 nM and (b) that formation of the above IAPP-GI oligomers likely depletes from the solution large amounts of the IAPP-GI species which are able to bind Aβ40 with low nanomolar affinity.

**Figure 6:**
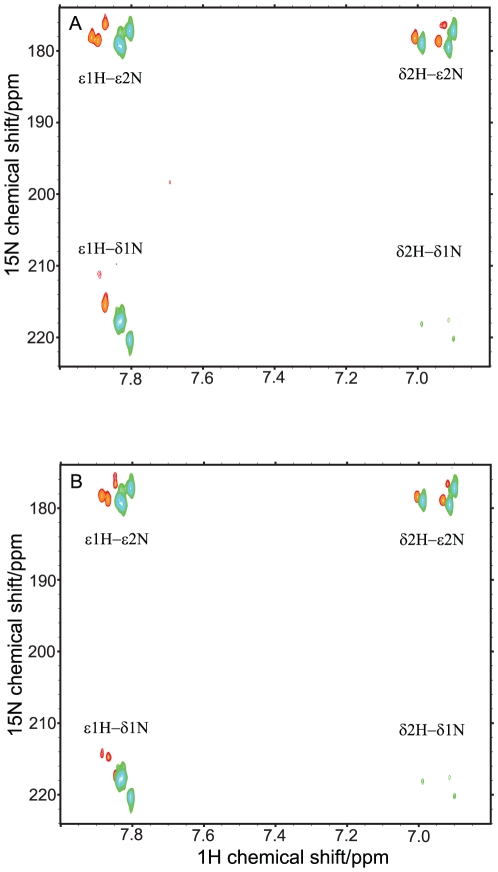
Influence of IAPP-GI binding on the protonation and tautomeric states of the histidines. A. Side chain^1^H-^15^N-HSQC spectra of Aβ40, in the absence (green) and presence (red) of IAPP-GI. Magnetization was transferred through an INEPT-based coherence transfer from ε1 and δ2 protons of histidine side chains to ε2 and δ1 nitrogen nuclei (see Figure 1B). The total duration of the INEPT was set to 27.8 ms. B. Comparison of side chain ^1^H-^15^N-HSQC spectra of Aβ40 (green) and Aβ42 (red). The cross-peak pattern of all the three histidine residues of Aβ (His6, His13 and His14) was observed.

To obtain further insights into the contribution of His13 and His14 for binding to IAPP-GI, side chain ^1^H-^15^N HSQC spectra in the free and IAPP-GI-interacting forms of Aβ40 were compared. The chemical shifts of the ε2 and δ1 nitrogens of the imidazole ring of histidine residues and their pattern of coupling to carbon-attached ε1 and δ2 protons are sensitive probes of their protonation, tautomeric and hydrogen bonding states [29]. As depicted in Figure 6, peaks of all three histidine side chains (including His6, which is not present in the backbone spectra) could be observed, and two of them, probably the consecutive His13 and His14, have very similar chemical shifts of ε2N, δ1N and ε1H. There is a large dispersion in the nitrogen dimension, indicating that the imidazole ring should be in an amphionic (non-protonated) state. This is consistent with the pH 7.3 of our sample, which is around one unit above the expected pKa of histidines. The chemical shifts of the ε2 and δ1 nitrogens are in the range of 177–180 and 218–220 ppm, respectively, suggesting that they may be involved in some form of H-bonds [30]. The observed pattern of coupling of ^15^N nuclei to ^1^H denote that the histidine side chains are largely populated in a tautomeric state in which ε2N is attached to a proton, and the weak intensity of the δ2H-δ1N peak suggest a small but still detectable ^3^J coupling between them [31]. After addition of IAPP-GI, the large dispersion in ^15^N dimension and the characteristic pattern of coupling between ^15^N and ^1^H nuclei remained unchanged. However, the following changes were observed: first, while all three ε2N chemical shifts were changed by a similar value of 0.8 ppm toward downfield, δ1N chemical shifts were affected more strongly and not in a uniform manner. The perturbation of the δ1N chemical shift was 4.7 and 6.5 ppm for two histidines and around 10 ppm for the third one, which is also severely broadened. Secondly, all the peaks experienced a significant degree of broadening upon addition of IAPP-GI. The intensity ratio for the ε2N correlations was between 20 and 30% for all the histidines of Aβ40. In contrast, for the δ1N correlations, it was 28 percent for one and only about 7 percent for the other two. Third, the weak ^3^J-based δ1N-δ2H peaks completely disappeared after addition of IAPP-GI. The direction of chemical shift deviation can be taken to suggest that, after interaction with IAPP-GI, δ1N of His13 and His14 spends more time in the protonated state and/or by average, has a smaller degree of solvent exposure [30]. The change in the protonation state cannot be caused by a variation of the sample pH, as the pH was accurately adjusted after addition of IAPP-GI. Instead, it is likely to originate from a change in the pKa of the histidines caused by binding-induced changes in the micro-environment. The significant broadening of the δ1N correlations might be attributed to a fast/intermediate exchange of Aβ40 between free and bound forms. To further investigate if these changes are caused by intermolecular interactions between Aβ40 and IAPP-GI, we measured the same spectra for the more aggregation-prone Aβ42. As displayed in Figure 6B, pure Aβ42 showed a pattern that is very similar to that of Aβ40 with IAPP-GI, further suggesting that the observed changes could be an effect of transient intermolecular interactions between Aβ and IAPP-GI.

**Figure 7:**
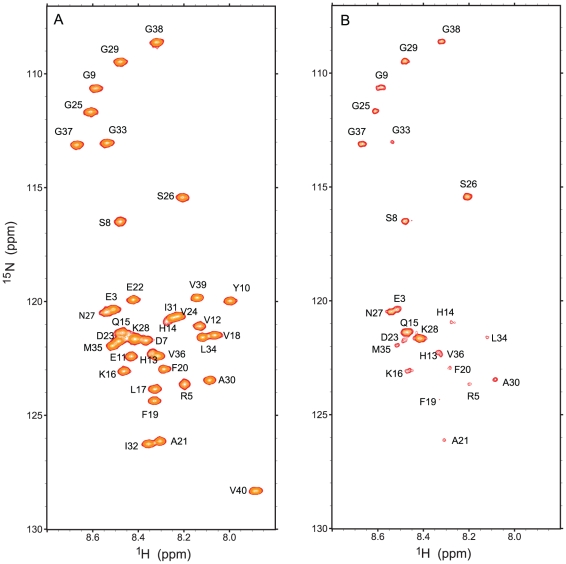
Probing the water-amide proton exchange rates through a CLEANEX-PM-FHSQC experiment. A. ^1^H-^15^N-FHSQC spectrum of free Aβ40. The peak intensities of the ^1^H-^15^N-FHSQC spectrum were used as reference to calculate the relative intensity of peaks due to chemical exchange. B. CLEANEX-PM-FHSQC spectrum, measured after a selective excitation of water protons and a following 100 ms mixing time during which chemical exchange between water and amide protons occurred variably for different residues of Aβ40.

**Figure 8:**
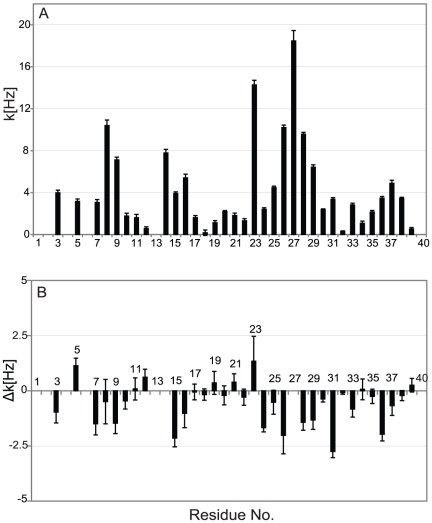
Initial water-amide proton exchange rates (k). A. Exchange rate, k, along the sequence of Aβ40 in the free form, measured through a CLEANEX-PM-FHSQC experiment. B. The change in exchange rate of Aβ40 residues after addition of a 16-molar excess of IAPP-GI. Residue numbers are indicated.

**Figure 9:**
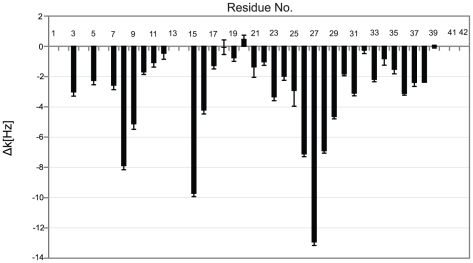
Difference between the initial water-amide proton exchange rates of Aβ42 and Aβ40 (Δk = Aβ42−Aβ40). The initial exchange rates were measured through a CLEANEX-PM-FHSQC experiment. Most residues show less solvent exposure in Aβ42 than Aβ40.

Next, we measured sequence specific water-amide proton exchange rates for Aβ40 in the absence and presence of IAPP-GI using CLEANEX-PM-FHSQC experiments [32]. The water-amide proton exchange rate is a valuable probe of the degree of exposure of the protein backbone to the solvent: a N-H group that is buried inside the protein or involved in a hydrogen bond, will be protected from exchange with solvent. In a CLEANEX-PM-FHSQC experiment, exposed parts of the backbone gain high signal intensity already at short mixing times due to efficient exchange with water, while for more protected groups the signal intensity only rises at longer mixing times. In the absence of IAPP-GI, Gly25, Ser26, Asn27, Lys28 and Gly29 of Aβ40 showed a strong increase in NMR signal intensity already at a mixing time of 100 ms (Figure 7). The solvent exposure of these residues was further corroborated by analysis of the initial exchange rate (Figure 8A): the highest exchange rates were observed for residues Ser8-Gly9, His14, Lys16, Asp23, Gly25–Gly29 and Gly37–Gly38. Interestingly, Gln15-Val24 and Ala30-Val36 have a high tendency to adopt α-helical [33] or β-strand [34] secondary structures, while the intervening region of Val24-Gly29 tends to form a loop [35]. Additionally, solid-state NMR studies of Aβ fibrils have verified that the region between residues 23 and 29 forms an exposed turn-like structure between two β-strands [36], [37]. Altogether, the observed profile of exchange rates suggests that the monomeric free form of Aβ40 transiently samples conformational states, in which residues 10–13, 17–22 and 30–36 are partially protected from exchange with solvent. Addition of IAPP-GI results in a decrease of the exchange rate of residues Asp7, Gly9, Gln15 and Lys16, as well as Val24–Val36 (Figure 8B). The reduced exchange rates suggest that these residues are less exposed to the solvent after IAPP-GI addition. Increased protection could be a result of an intermolecular interaction between IAPP-GI and Aβ40, or alternatively caused by an IAPP-GI induced change in the conformation or assembly state of Aβ40. Support for the influence of intermolecular interactions comes from a comparison of exchange rates in Aβ40 and Aβ42. In Aβ42, which has a higher tendency for self-association, the exchange rates are reduced in regions (Figure 9) that are also affected upon addition of IAPP-GI to Aβ40 (Figure 8B).

A recent analysis of the Aβ-IAPP and Aβ-IAPP-GI interaction interface using synthetic peptides and peptide arrays has shown that the “hot spot regions” of the Aβ-IAPP and Aβ-IAPP-GI interfaces are localized predominantly within the two regions which are converted into β-strands in amyloid fibrils [38]. These studies have also suggested that the hydrophobic C-terminal part of Aβ (residues 29–40) binds IAPP (and IAPP-GI) with a 10-fold higher affinity as compared to the N-terminal part (residues 1–28), which binds IAPP with low µM affinity [38], and that the region between Phe19 and Glu22 was the shortest residue stretch in the N-terminal part of Aβ required for low µM binding, while no evidence for a direct interaction of the Aβ region between His13 and Leu17 was observed [38]. Our NMR chemical shift perturbation data assign now an important role in the Aβ-IAPP-GI interaction to residues His13 to Leu17, the region of Aβ that is important for binding to ganglioside micelles [39]. At the same time, the water-amide proton exchange data of Aβ40 in the absence and presence of IAPP-GI suggest that in addition to the N-terminal region, residues in the C-terminal part are also involved in the interaction. Taking all these findings into consideration, we propose the following model for the Aβ40-IAPP-GI interaction: The C-terminal part of Aβ is directly involved in intermolecular interaction with IAPP-GI. This intermolecular interaction induces a conformational change in the N-terminal part of Aβ, which is favored by the high propensity of the N-terminal region of Aβ40 to assume secondary structure [33], [34]. The conformational transition then leads to changes in pKa and protonation state of the histidine residues. Since the IAPP-GI peptide has a high degree of sequence similarity to Aβ, the Aβ-IAPP-GI interaction might represent some of the initial steps in self-association of Aβ molecules. In fact, the proposed model for interaction between Aβ and IAPP-GI has some similarities with a recently published structure of Aβ oligomers, where the N- and C-terminal parts of Aβ are involved in intra- and inter-molecular interactions, respectively [40].

The prominent loss of NMR signal intensities in the ^1^H-^15^N HSQC spectra of Aβ40 and Aβ42 upon increasing concentrations of IAPP-GI points to a IAPP-GI dependent conversion of Aβ to an NMR-invisible state, i.e. a large and/or non-homogeneous aggregate state with slow tumbling and/or significant inhomogeneity that broaden its signals beyond experimental detection level. Because the NMR-visible signals of IAPP-GI are reduced at the same time, it is most likely that aggregates are formed that comprise both Aβ and IAPP-GI [18]. In agreement with their intrinsic aggregation propensities, the aggregation is more evident in the case of Aβ42 than Aβ40. As it has previously been shown that IAPP-GI significantly lowers Aβ-mediated toxicity when both are mixed in micromolar concentrations, i.e. similar to the conditions used in the current study, our data suggests that the aggregate state of Aβ induced in the presence of IAPP-GI is not a toxic “on-pathway” aggregate, but a nontoxic “off-pathway” aggregate which is consistent with previous findings [18]. A similar redirection of Aβ and α-synuclein into off-pathway oligomers was previously reported for the polyphenol (-)-epigallocatechin gallate [41], suggesting a similar toxicity lowering mechanism of IAPP-GI and (-)-epigallocatechin gallate, at least at high inhibitor/peptide concentrations. Finally, our studies provide evidence that the interaction between Aβ and IAPP-GI leads to a conformational change in the region Aβ (13–17) that might be related to the inhibitory effect of IAPP-GI on Aβ “on-pathway” aggregation and cell toxicity.

## Materials and Methods

### Materials

^15^N-uniformyl labeled Aβ1-40 and Aβ1-42 was purchased from rPeptide, and dissolved in 10 mM NaOH solution at 2 mg/mL concentration [8]. Synthetic IAPP-GI was synthesized as previously described [13] and dissolved in pure 1,1,1,3,3,3,-hexafluoroisopropanol (HFIP) at a concentration of 5 mg/mL. N-terminal fluorescein labeled Aβ40 was prepared as described for the labeled IAPP segments [38]. HFIP was obtained from Fluka. Before titration experiments, the solvent (HFIP) was evaporated under N_2_ gas and phosphate buffer was used to dissolve dried IAPP-GI.

### NMR Spectroscopy

All NMR spectra were recorded at 278 K with a sample of 30 µM ^15^N-uniformly labeled Aβ40 or Aβ42, with or without the specified concentration of non-labeled IAPP-GI, in 20 mM sodium phosphate buffer at pH 7.3 (adjusted after adding peptides). Chemical shift referencing at this temperature was made with respect to the external 4,4-dimethyl-4-silapentane-1-sulfonic acid (0.0 ppm). All NMR spectra were processed and analyzed with NMRPipe and Sparky.

The interaction of IAPP-GI with Aβ40 and Aβ42 was assessed through a series of ^1^H-^15^N HSQC spectra of Aβ in the presence of 0, 2, 4, 8 and 16 molar excess of IAPP-GI for Aβ40 and 0, 1, 2, 4 and 8 molar excess of IAPP-GI for Aβ42. Titrations were made on the same peptide sample, and temporal stability of peptide solutions were checked with 1D ^1^H-NMR spectra measured before and after each NMR experiment. The spectra were measured on a Bruker Avance 600 MHz spectrometer equipped with a triple-resonance room temperature probe. The time domain data contained 1 K and 220 complex data points in t2 and t1, respectively. The 90°-shifted squared sine bell function was used as apodization function in both dimensions, yielding a final matrix of 8K*512 data points. The peak assignments were obtained from the literature, and their deviation from the original position was monitored as a function of IAPP-GI/Aβ molar ratio. The average chemical shift deviation was calculated as the root square of (ΔN/5)∧2+(ΔH)∧2, where ΔN and ΔH were the chemical shift difference of N and H^N^ resonances compared to the control experiment. The first (IAPP-GI∶Aβ ratio of 0) and last (IAPP-GI∶Aβ ratio of 16) experiments were re-measured on the higher field Bruker AMX 800 MHz, equipped with a cryogenic probe to confirm the observed chemical shift perturbations. In a control experiment, Aβ40 was similarly titrated with phosphate buffer after dissolving the dried HFIP (without IAPP-GI) under N_2_ gas.

^1^H-^15^N HSQC spectra of the side chains of histidines (H6, H13 and H14) were acquired on a Bruker AMX 600 MHz spectrometer equipped with a triple-resonance cryogenic probe. The sample was 30 µM Aβ40, with or without a 16 molar excess of IAPP-GI. A 30 µM sample of Aβ42 was also measured. The INEPT transfer delay was selected as 27.8 ms, to obtain the coherence transfer from the ε1H and δ2H protons to the ε2N and δ1N through the ^2^J coupling constant (Figure 1B). The carrier and SWH values were 5.00 ppm and 6010 Hz in the direct dimension, and 185.31 ppm and 9728 Hz in the indirect dimension. The experimental matrix was 2K*256 complex data points, which after apodization through a 90°-shifted squared sine bell function in both dimensions was transformed to a final matrix of 8K*512 data points.

The solvent accessibility of the Aβ40 backbone in the free and possibly IAPP-GI-bound forms was evaluated through measurement of exchange rates between water and NH protons. To this end, CLEANEX-PM-FHSQC experiments were recorded on a Bruker Avance 600 MHz spectrometer equipped with a triple-resonance room temperature probe. In a CLEANEX-PM-FHSQC experiment, a selective water excitation is followed by a mixing time (τ_m_) of durations 4, 8, 16, 24, 32, 48, 75, 100, 200 and 500 ms, during which chemical exchange between water and NH protons takes place. The intensity of peaks as a function of mixing time was fitted to the following equation:
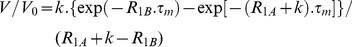
(1)where *V_0_* is the intensity of the peak in a control FHSQC experiment, *k* is the normalized rate constant related to the forward exchange rate constant between water and NH protons, and *R_1A_* and *R_1B_* are apparent longitudinal relaxation rates for protein and water [32].

## Supporting Information

Figure S1 **1D ^1^H NMR spectra of Aβ42 with or without IAPP-GI.** The methyl and aromatic regions of the spectra are shown in (A) and (B), respectively. The spectra were measured in the presence of 0 (black), 1 (red), 2 (orange), 4 (green) and 8 (blue) molar excess of IAPP-GI. Chemical shifts were calibrated on the basis of an external DSS reference and the temperature was set to 5°C. Peaks marked as * are observed exclusively after addition of IAPP-GI, and their relative intensities are taken to follow the rise of NMR-visible IAPP-GI concentration.
(EPS)

Figure S2 **Loss of the peak intensities in ^1^H-^15^N-HSQC spectra of ^15^N-labeled Aβ42 upon addition of non-labeled IAPP-GI.** IAPP-GI/Aβ42 molar ratios varied between 0, 1, 2, 4 and 8, as indicated. The intensity ratios were calculated relative to the intensities of free Aβ42 spectrum.
(EPS)

Figure S3 **Dynamic light scattering of Aβ40.** In addition to a small monomeric peak around 1.6 nm, two oligomeric peaks are observed at around 30 and 150 nm. Note that the intensity of the peaks does not reflect the relative amount of each species, as large species scatter light more intensely than the small NMR-visible monomeric species at 1.6 nm.
(EPS)

Figure S4 **Far-UV CD concentration dependence study of the conformation of IAPP-GI.** CD spectra of IAPP-GI were measured in aqueous buffer, pH 7.4 (1% HFIP), at peptide concentrations between 500 nM and 50 µM, as indicated.
(EPS)

Figure S5 **Aβ40-IAPP-GI interaction as determined by fluorescence spectroscopy.** N-terminal fluorescein labelled Aβ40 (Fluos-Aβ40) (1 nM) was titrated with increasing amounts of IAPP-GI as indicated. (A) The binding curve of Fluos-Aβ40-IAPP-GI interaction obtained using concentrations of IAPP-GI between 1–500 nM is shown (fluorescence emission at 522 nm). (B) The fluorescence emissions at 522 nm of the different mixtures of Fluos-Aβ40 with IAPP-GI made using concentrations of IAPP-GI between 1 nM and 10 µM are shown. Fluorescence of Fluos-Aβ40 alone was subtracted.
(EPS)

Methods S1 **Dynamic Light Scattering.**
(DOC)
