# Supplementary material for: Interaction between Amyloid Beta Peptide and an Aggregation Blocker Peptide Mimicking Islet Amyloid Polypeptide
Source: PLoS One. 2011 May 25;6(5):e20289. doi: 10.1371/journal.pone.0020289 (PMC3102090; doi:10.1371/journal.pone.0020289)
Supplement: Methods S1 — Dynamic Light Scattering. (DOC) [file pone.0020289.s006.doc]

**Methods.**

**Dynamic Light Scattering**

DLS experiment was performed at 5 °C on a DynaPro Titan (Wyatt Technology Corp., CA) instrument, with a laser of 827.08 nm and scattering angle of 90°. Aβ was initially dissolved in 10 mM NaOH at 2 mg/mL concentration, then brought into a solution of pH 7.2 buffered with 20 mM sodium phosphate with a final Aβ concentration of 30 μM (same concentration as the NMR experiment). Then, peptide solution was centrifuged at 16100 g for 30 minutes and supernatant was immediately measured. DLS measurement consisted of twelve 20-s long acquisitions, with 1 minute waiting time between them, and was done in duplicate. Refractive index (RI) of the solution was set at 1.333 at 589 nm and 20 °C, and the RI at the studied wavelength was obtained through Cauchy equation, with a coefficient of 3119 nm^2^. The viscosity was 1.019 cp at 20 °C and the temperature-dependent variations were calculated by an aqueous model. The size distribution was determined by a constrained regularization method.

**Far-UV CD spectroscopy**

Far-UV CD measurements were carried out with an AVIV 202SF spectropolarimeter as previously described [18]. The measurements were performed in 10 mM sodium phosphate buffer, pH 7.4 containing 1% HFIP and at peptide concentrations between 500 nM and 50 μM as indicated. IAPP-GI stock solutions in HFIP were used.

**Fluorescence spectroscopy studies**

Fluorescence measurements were performed using a Spex Fluorolog 2 fluorescence spectrophotometer and synthetic N-terminal fluorescein labeled Aβ40 at room temperature as previously described [18].
